# Myocardial dysfunction and chronic heart failure in patients with long-lasting type 1 diabetes: a 7-year prospective cohort study

**DOI:** 10.1007/s00592-013-0455-0

**Published:** 2013-01-30

**Authors:** Ewa Konduracka, Grazyna Cieslik, Danuta Galicka-Latala, Pawel Rostoff, Artur Pietrucha, Pawel Latacz, Grzegorz Gajos, Maciej T. Malecki, Jadwiga Nessler

**Affiliations:** 1 Department of Coronary Disease, Jagiellonian University School of Medicine, John Paul II Hospital, Prądnicka 80, 31-202 Krakow, Poland; 2 Diabetology Consulting Unit, Centre for Treatment of Civilization-Induced Diseases, Krakow, Poland; 3 Department of Metabolic Diseases, Jagiellonian University School of Medicine, Krakow, Poland

**Keywords:** Type 1 diabetes, Myocardial dysfunction, Heart failure, Incidence, Prevalence, NT-proBNP

## Abstract

The aim of the study is to evaluate the prevalence and incidence of myocardial dysfunction (MD) and heart failure (HF) in long-lasting (≥10 years) type 1 diabetes without cardiovascular disorders or with hypertension or coronary heart disease (CHD). The study included *1,685 patients with* type 1 diabetes (mean baseline age, 51 years; diabetes duration, 36 years). In all patients, echocardiography was performed, NT-proBNP levels were measured, and clinical symptoms were evaluated. A 7-year follow-up was conducted to monitor systolic and diastolic manifestations of MD and HF. At the end of the follow-up period, the prevalence of HF in the entire group was 3.7 %, and the incidence was 0.02 % per year. The prevalence of MD was 14.5 % and the incidence –0.1 % per year. MD and HF were observed only in hypertensive or CHD patients. At baseline, subjects with diastolic HF constituted 85 % of the HF population and those with systolic HF the remaining 15 %. Baseline HF predictors included age, diabetes duration, HbA_1c_ levels, CHD, systolic blood pressure >140 mmHg, and GFR <60 mL/min/1.73 m^2^. In patients with type 1 diabetes, MD and HF occurred only when diabetes coexisted with cardiovascular disorders affecting myocardial function. The prevalence and incidence of HF in patients with hypertension and CHD were relatively low. While the cause of this observation remains uncertain, it could probably be explained, at least partially, by the cardioprotective effect of concomitant treatment.

## Introduction

The prevalence of heart failure (HF) in the general population is estimated at 1–4 %, depending on the age group. According to the epidemiological studies, from 12 to 22 % of diabetic patients suffer from HF [1–9].

A number of independent risk factors for the development of HF have been identified in patients with diabetes. Apart from metabolic disturbances related to hyperglycaemia, the two most common risk factors for the development of HF are coronary heart disease (CHD) and hypertension, which are more prevalent in patients with diabetes than in the general population [1–10].

Myocardial dysfunction (MD) is a clinically asymptomatic state, which may precede or coexist with HF symptoms. Early diagnosis of MD is vitally important because specific therapy applied at this stage can effectively delay the actual onset of HF. However, the prevalence of MD and HF in diabetic patients has not been extensively studied. The majority of studies to date have focused only on type 2 diabetes or did not distinguish between type 1 and type 2 diabetes. Moreover, they did not differentiate between systolic and diastolic manifestations of MD and HF [1–10].

Determining the effect of metabolic disturbances associated with type 1 diabetes on cardiac function still remains a challenge in the era of modern intensive insulin therapy. The potential effect of hypertension and CHD on the prevalence of MD and HF in type 1 diabetes also requires further investigation.

The aim of the current study was to examine the prevalence and incidence of MD and HF in long-lasting (≥10 years) type 1 diabetes without cardiovascular comorbidities or with concomitant hypertension and CHD.

## Methods

Our research was designed as a prospective cohort study.

### Cohort

We identified a cohort from the registries of diabetes clinics in 5 Polish counties and of the Department of Metabolic Diseases at the Jagiellonian University School of Medicine, Krakow, which is a reference centre for diabetes care in south-eastern Poland. We also had access to the medical records of all hospitalized diabetic patients in the above facilities. All 1,856 subjects were recruited between 1999 and 2004. The cohort included consecutive patients that had been diagnosed at the Department of Coronary Disease at the Jagiellonian University. It corresponds to the number of type 1 diabetic patients who agreed to participate in the study during that period.

### Inclusion criteria

1. Patients over 18 years of age with type 1 diabetes (insulin therapy from the beginning of the disease; diabetes diagnosed before the age of 30),
2. Diabetes duration ≥10 years.

### Exclusion criteria (129 individuals excluded)

1. Comorbidities other than hypertension and CHD that can affect myocardial function: pulmonary disorders with dyspnoea (*n* = 53), severe rheumatic valve diseases (*n* = 42), a history of myocarditis (*n* = 10), systemic diseases with cardiac involvement (*n* = 4)
2. Difficulty in assessing diastolic function (permanent atrial fibrillation, cardiac pacemaker); *n* = 10
3. Refusal to give informed consent (*n* = 10).

### Clinical examination

All participants underwent baseline subjective and objective clinical examination. Hypertension was defined as ≥140/90 mmHg (at least on two separate measurements) or hypotensive therapy initiated due to elevated blood pressure. Cardiac autonomic neuropathy (CAN) was diagnosed according to the applicable standards [11, 12].

### Laboratory measurements

Baseline serum N-terminal pro-B-type natriuretic peptide (NT-proBNP) levels were determined with an electrochemiluminescence assay (Elecsys, Roche Diagnostic, France) in one central laboratory. The normal value for healthy individuals aged <75 years was <125 pg/ml. Other biochemical tests were also performed as presented in Tables 1, 2, 3.

**Table 1:** Characteristics for the subjects without hypertension and without coronary disease (subgroup A) who completed the study, at baseline and at the 7 year of follow-up

Parameter	Type 1 diabetes		*p* value
	Baseline	At the 7 year of follow-up	
	*n* = 393	*n* = 390	
Sex/male *n* (%)	194 (49.4)	191 (49.0)	0.91
Age* (years)	34.8 ± 7.9	41.8 ± 7.9	0.45
BMI* (kg/m^2^)	25.4 ± 2.3	26.2 ± 2.5	0.05
HbA1c* (%)	7.8 ± 1.4	7.4 ± 1.3	0.05
Diabetes duration* (years)	26.4 ± 5.6	33.4 ± 5.6	0.32
Any diabetic retinopathy *n* (%)	106 (27)	109 (28)	0.90
CAN *n* (%)	4 (1.0)	5 (1.3)	0.99
GFR* (ml/min/1.73 m^2^)	110.0 ± 3.5	112.0 ± 3.4	0.09
Systolic BP* (mmHg)	125.2 ± 12.6	127.2 ± 11.8	0.07
Diastolic BP* (mmHg)	74.3 ± 11.5	75.4 ± 12.2	0.08
LDL-ch* (mmol/l)	3.1 ± 1.2	3.0 ± 0.8	0.05
Triacylglycerol* (mmol/l)	1.1 ± 0.5	1.0 ± 0.5	0.17
HDL-ch* (mmol/l)	1.4 ± 0.4	1.5 ± 0.3	0.65
Statin therapy *n* (%)	158 (40.2)	217 (55.6)	<0.0001

*CAN* cardiac autonomic neuropathy, *BP* blood pressure, *GFR* glomerular filtration rate

* Mean values ± SD

**Table 2:** Characteristics of type 1 diabetic subjects with hypertension (subgroup B) who completed the study, at baseline and at the 7 year of the follow-up period

Parameter	Type 1 diabetes		*p* value
	Baseline	At the 7 year of follow-up	
	*n* = 1,199	*n* = 1,199	
Sex/male *n* (%)	605 (50.5)	605 (50.5)	1.00
Age* (years)	57.7 ± 4.3	64.7 ± 4.3	0.40
BMI* (kg/m^2^)	25.2 ± 3.1	27.1 ± 2.6	0.05
HbA1c (%)	8.5 ± 1.4	8.4 ± 1.3	0.65
Diabetes duration* (years)	38.4 ± 15.2	45.4 ± 15.2	0.30
Any diabetic retinopathy *n* (%)	959 (80)	963 (80.3)	0.86
Sensorimotor neuropathy *n* (%)	72 (6)	84 (7)	0.85
CAN *n* (%)	42 (3.5)	44 (3.7)	0.83
GFR* (ml/min/1.73 m^2^)	77.0 ± 15.1	70.1 ± 15.1	0.05
Albuminuria *n* (%)	88 (7.3)	92 (7.7)	0.76
Systolic BP* (mmHg)	135.5 ± 8.3	129.3 ± 8.9	<0.0001
Diastolic BP* (mmHg)	87.3 ± 14.4	83.1 ± 13.2	0.008
LDL-ch* (mmol/l)	3.3 ± 0.4	2.8 ± 0.4	0.001
Triacylglycerol * (mmol/l)	1.3 ± 0.3	0.9 ± 0.2	<0.0001
HDL-ch* (mmol/l)	1.3 ± 0.3	1.4 ± 0.3	0.09
ACE-i *n* (%)	1,005 (83.8)	1,010 (84.2)	0.78
ARA *n* (%)	90 (7.5)	85 (7.1)	0.69
Ca-blocker *n* (%)	715 (59.6)	890 (74.2)	<0.0001
β-blocker *n* (%)	428 (35.7)	433 (36.1)	0.83
Aspirin *n* (%)	1,180 (98.4)	1,189 (99.2)	0.09
Statin *n* (%)	1,100 (91.7)	1,190 (99.2)	<0.0001
Fibrate *n* (%)	23 (1.9)	24 (2.0)	0.88
Diuretic *n* (%)	252 (21.0)	261 (21.8)	0.65

*ARA* angiotensin receptor antagonist, *Ca*-*blocker* calcium channel blocker, *CAN* cardiac autonomic neuropathy, *BP* blood pressure, *GFR* glomerular filtration rate

* Mean values ± SD

**Table 3:** Characteristics of type 1 diabetic subjects with hypertension and CHD (subgroup C) who completed the study, at baseline and at the 7 year of follow-up period

Parameter	Type 1 diabetes		*p* value
	Baseline	At the 7 year of follow-up	
	*n* = 93	*n* = 96	
Sex/male *n* (%)	53 (57.0)	56 (58.3)	0.85
Age* (years)	58.7 ± 4.3	65.7 ± 4.3	0.40
BMI* (kg/m^2^)	25.2 ± 3.1	27.1 ± 2.6	0.05
HbA1c (%)	8.5 ± 1.4	8.4 ± 1.3	0.65
Diabetes duration* (years)	38.4 ± 15.2	45.4 ± 15.2	0.30
Any diabetic retinopathy *n* (%)	93 (100)	96 (100)	1
Sensorimotor neuropathy *n* (%)	9 (10)	12 (12)	0.88
CAN *n* (%)	40 (43.0)	43 (44.8)	0.81
GFR* (ml/min/1.73 m^2^)	77.0 ± 15.1	70.1 ± 15.1	0.06
Albuminuria *n* (%)	85 (91.4)	90 (93.8)	0.54
Systolic BP* (mmHg)	135.5 ± 8.3	129.3 ± 8.9	<0.0001
Diastolic BP* (mmHg)	87.3 ± 14.4	83.1 ± 13.2	0.008
LDL-ch* (mmol/l)	3.3 ± 0.4	2.8 ± 0.4	0.001
Triacylglycerol * (mmol/l)	1.3 ± 0.3	0.9 ± 0.2	<0.00001
HDL-ch* (mmol/l)	1.3 ± 0.3	1.4 ± 0.3	0.09
Previous MI *n* (%)	17 (18.3)	25 (26.0)	0.20
Revascularization *n* (%)	40 (43.0)	58 (60.4)	0.017
ACE-i *n* (%)	82 (88.2)	82 (85.4)	0.58
ARA *n* (%)	8 (8.6)	11(11.5)	0.51
Ca-blocker *n* (%)	82 (88.2)	80 (83.3)	0.34
β-blocker *n* (%)	86 (92.5)	86 (89.6)	0.49
Aspirin *n* (%)	93 (100.0)	96 (100.0)	1.00
Statin *n* (%)	93 (100.0)	96 (100.0)	1.00
Fibrate *n* (%)	6 (6.5)	5 (5.2)	0.96
Diuretic *n* (%)	35 (37.6)	55 (57.3)	0.007
Nitrate *n* (%)	80 (86.0)	82 (85.4)	0.91
Aldosterone antagonist *n* (%)	2 (2.2)	3 (3.1)	0.97

*ARA* angiotensin receptor antagonist, *Ca*-*blocker* calcium channel blocker, *CAN* cardiac autonomic neuropathy, *MI* myocardial infarction, *GFR* glomerular filtration rate, *BMI* body mass index

* Mean values ± SD

### Echocardiography

Complete echocardiography was performed using the Simens Sequoia C 512 ECHO unit equipped with a multi-frequency, harmonic transducer (2.5–4 MHz). The average values of three consecutive measurements were recorded. All patients were examined by the same operator.

HF with preserved left ventricular (LV) ejection fraction (EF) (HFPEF) or diastolic heart failure was defined as the presence of:

1. Signs or symptoms of HF
2. Normal or mildly abnormal LV systolic function (EF ≥ 50 %)
3. Evidence of LV diastolic dysfunction.

All three criteria had to be met to diagnose HFPEF [13, 14]Heart failure with reduced LVEF (HFREF) was defined as the presence of:

1. Signs or symptoms of HF
2. Evidence of LV systolic dysfunction.

Both criteria had to be met to diagnose HFREF [13, 14].

Diastolic LV dysfunction was diagnosed when: [14]

- E/E′ > 15 or
- 8 < E/E′ < 15 and the serum level of NT-proBNP > 220 pg/ml or
- 8 < E/E′ < 15 and any of the following criteria:

- E/A < 1.0 and DT > 200 ms < 50 years
- E/A < 0.5 and DT > 280 ms > 50 years (impaired relaxation)
- E/A = (1.0–2.0)

and at least two (jointly) of the following criteria:

- S/D < 1 or Ar ≥ 35 cm/s or E′ < A′ (pseudonormalization)
- E/A > 2.0 and DT < 150 ms, E′ < A′ (restriction)

(d) Left atrial volume index > 40 ml/m^2^.

Systolic LV dysfunction was diagnosed when the LVEF was <50 % in echocardiography.

### Exercise treadmill test, perfusion scintigraphy

The remaining 1,727 subjects underwent an exercise treadmill test (ETT) or perfusion scintigraphy (when ETT was contraindicated or inconclusive) to exclude CHD. Then, coronary angiography was performed in 102 patients with the positive results of ETT or scintigraphy. Significant CHD was diagnosed if the luminal diameter of the vessel was reduced by ≥50 %.

### Study subgroups

**Fig. 1:**
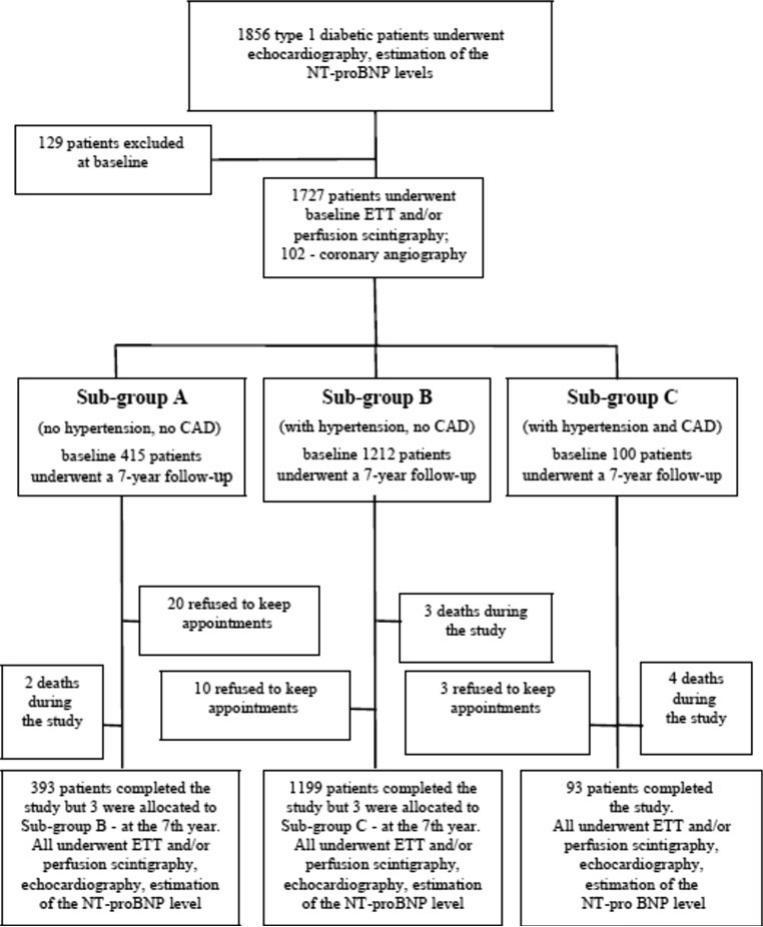
Flow diagram describing the cohort changes from enrolment to study completion

After baseline assessment, the study cohort was divided into three subgroups (A, B, and C) to evaluate the effect of diabetes on cardiac function in patients without hypertension and CHD (subgroup A), in patients with concomitant hypertension (subgroup B), and in those with significant CHD (subgroup C**)** (Fig. 1).

All patients with significant CHD had concomitant hypertension.

### Follow-up

Study subjects were followed up for 7 years. All diagnostic procedures were repeated at 7 years or earlier if necessary. During the follow-up, coronary angiography was performed in 26 patients (3 new cases of stable CHD with positive ETT; recurrent stable angina in 15 patients with previously diagnosed CHD; acute coronary syndromes in 8 patients with previously diagnosed CHD).

All participants were treated in compliance with the current guidelines on cardiac and diabetes care.

A total of 42 patients were lost to follow-up:

1. Patients unable to keep appointments (*n* = 33; without MD and HF). There were no significant clinical differences in demographic parameters, NT-proBNP levels, and the results of echocardiography between patients who dropped out from the study and those who completed the study (data not shown).
2. Deaths during the study (all patients without MD and HF before death): hypoglycaemia (*n* = 2), pulmonary embolism (*n* = 2), unknown aetiology of sudden death (*n* = 1), stroke (*n* = 2), cancer (*n* = 2).

At 7 years, there were 3 new cases of hypertension diagnosed in subgroup A. These participants were subsequently moved to subgroup B. At 7 years, there were also 3 new cases of significant CHD diagnosed in subgroup B. These participants were subsequently moved to subgroup C.

A total of 1,685 subjects with type 1 diabetes completed the study (men, 50.6 %; baseline mean age, 51.2 ± 10.3 years; mean HbA_1c_, 8.2  ± 1.2 %). Only patients who fully complied with the protocol were included in the final analysis. The number of subjects in particular subgroups who completed the study was as follows:

- subgroup A: baseline—393, at 7 years—390
- subgroup B: baseline—1,199, at 7 years—1,199
- subgroup C: baseline—93, at 7 years—96

All participants gave their written informed consent. The study was approved by the Ethical Review Committee and the study protocol complied with the Helsinki Declaration.

### Study end-points

The primary end-point was the prevalence and incidence of MD and HF in the study population. The secondary end-point were NT-proBNP levels. The outcomes were evaluated independently by an adjudication committee consisting of experienced cardiologists. The committee was blinded to concomitant disorders (hypertension, CHD) and metabolic control in the study population.

### Statistical analysis

Statistical analysis was performed using the STATISTICA 7.0 PL software. All continuous variables were expressed as mean ± standard deviation, and categorical variables were expressed as percentages.

The Mann–Whitney *U* test was used to compare normally and non-normally distributed continuous variables. The analysis of variance (ANOVA) or the Kruskal–Wallis test were applied where appropriate.

The χ^2^ test was used to evaluate the differences in categorical variables between the subgroups. All statistical tests were two-sided. The relationships between continuous variables were assessed by the Spearman’s rank correlation. A multiple regression analysis was used for baseline estimation of the predictors of HF prevalence, due to the small incidence of new HF cases during the 7-year follow-up (5 persons). Statistical significance was accepted at *p* < 0.05.

Prevalence was defined as the total number of HF cases at baseline and at 7 years, calculated per 1,000 subjects. Incidence was defined as the number of new HF cases in the study population per year over the 7-year follow-up period, calculated per 1,000 subjects.

## Results

The characteristics of the subgroups are summarized in Tables 1, 2, 3. All patients were treated with the model of multiple (4 or more) insulin injections.

### Echocardiography and serum NT-proBNP levels

Patients in subgroup A (no hypertension or CHD) had normal echocardiographic parameters and NT-proBNP levels. At baseline and at 7 years, MD was observed only in patients with hypertension and CHD (subgroups B and C).

Diastolic dysfunction was the most common abnormality and was observed in 15.6 % of the patients with hypertension and CHD.

The prevalence of MD (both systolic and diastolic manifestations) in the entire group was 14.5 %, and the incidence at 7 years was 0.01 % per year. During the 7-year follow-up, 3 new cases of MD were diagnosed.

The results of echocardiography and NT-proBNP levels at baseline and at 7 years are summarized in Table 4.

**Table 4:** Results of echocardiography and the NT-proBNP levels in the respective subgroups at baseline and at the 7 year of follow-up

Parameter*	Subgroup A		*p* value	Subgroup B		*p* value	Subgroup C		*p* value
	Baseline	At the 7 year of follow-up		Baseline	At the 7 year of follow-up		Baseline	At the 7 year of follow-up	
	*n* = 393	*n* = 390		*n* = 1,199	*n* = 1,199		*n* = 93	*n* = 96	
LVEF (%)**	64 ± 4.4	65 ± 4.3	0.48	64 ± 4.0	61.2 ± 3.8	0.40	58.1 ± 8.7	52.1 ± 6.4	0.04
LAVI (ml/m^2^)^†^	22.9 ± 5.0	23.1 ± 2.6	0.58	27.2 ± 2.3	31.3 ± 2.2	0.05	28.5 ± 1.9	31.5 ± 2.2	0.02
DT (ms)^††^	193.9 ± 10.9	196.8 ± 11.2	0.09	225.6 ± 43.4	230.0 ± 46.5	0.05	250.2 ± 64.7	267.7 ± 61.3	0.02
E/A ratio^#^	1.5 ± 0.2	1.3 ± 0.2	0.67	1.3 ± 0.4	1.2 ± 0.3	0.09	1.1 ± 0.6	0.9 ± 0.4	0.04
S/D ratio^##^	1.2 ± 0.3	1.1 ± 0.2	0.12	1.4 ± 0.4	1.2 ± 0.3	0.08	1.5 ± 0.7	1.1 ± 0.1	0.01
Ar (cm/s)^≠^	22.7 ± 4.7	23.7 ± 3.7	0.45	23.8 ± 3.8	24.4 ± 3.6	0.43	27.1 ± 4.4	29.1 ± 4.8	0.001
E/E′ ratio^‡^	5.5 ± 1.2	5.6 ± 1.0	0.70	7.5 ± 2.0	8.1 ± 2.1	0.08	8.8 ± 2.83	9.5 ± 2.2	0.001
NT-proBNP (ng/ml)^∫^	63.5 ± 18.4	69.0 ± 16.4	0.08	120.0 ± 52.1	132 ± 49.0	0.04	530.3 ± 129.2	690.5 ± 121.5	0.0001

* Mean values ± SD

Comparison between the respective subgroups: A versus B, A versus C, B versus C

** *p* = 0.56, *p* = 0.02, *p* = 0.03

^†^ *p* = 0.03, *p* = 0.01, *p* = 0.06

^††^ *p* = 0.01, *p* = 0.001, *p* = 0.56

^#^ *p* = 0.40, *p* = 0.04, *p* = 0.07

^##^ *p* = 0.50, *p* = 0.04, *p* = 0.08

^≠^ *p* = 0.06, *p* = 0.02, *p* = 0.01

^‡^ *p* = 0.03, *p* = 0.001, *p* = 0.001

^∫^ *p* = 0.01, *p* = 0.0001, *p* = 0.001

### Chronic heart failure

At the end of the follow-up, the prevalence of HF (both HFPEF and HFREF) was 3.7 % and the incidence was 0.02 % per year (only subgroups B and C).

At baseline, patients with HFPEF constituted 85 % of the HF population, while those with HFREF the remaining 15 %. During the follow-up, 1 new case of HFPEF and 4 new cases of HFREF were diagnosed.

The baseline predictors of prevalent HF are summarized in Table 5. Cardiac autonomic neuropathy, sensorimotor neuropathy, and any diabetic retinopathy were not found to be independent predictors of HF in a multivariate logistic regression analysis.

**Table 5:** Baseline predictors of prevalent heart failure in type 1 diabetic patients

Predictor	Women			Men		
	OR	95 % CI	*p* value	OR	95 % CI	*p* value
Univariate analysis						
Age (per year)	1.12	1.04–1.20	0.003	1.06	1.01–1.12	0.015
GFR < 60 ml/min/1.73 m^2^	3.17	1.41–7.11	0.005	7.06	3.19–15.59	0.000001
Significant CHD	2.88	1.14–7.48	0.04	3.40	1.37–8.41	0.008
Any diabetic retinopathy	12.34	1.65–92.12	0.01	2.49	1.01–6.28	0.005
Diabetes duration (per year)	1.07	1.04–1.11	0.0001	1.08	1.04–1.12	0.0001
Systolic BP > 140 mmHg	2.31	1.01–5.69	0.005	4.48	1.52–13.15	0.006
Diastolic BP > 90 mmHg	3.66	1.57–8.23	0.029	–	–	–
HbA1c (%)	1.61	1.25–2.07	0.0002	1.45	1.14–1.85	0.003
Triacylglycerol (mmol/l)	3.13	1.30–7.63	0.011	–	–	–
Albuminuria	–	–	–	4.94	2.23–10.96	0.00009
Multivariate analysis						
Age (per year)	1.05	1.01–1.14	0.04	1.01	1.01–1.08	0.04
Diabetes duration (per year)	1.06	1.02–1.11	0.006	1.06	1.02–1.11	0.006
Significant CHD	2.50	1.01–7.56	0.04	3.04	1.13–8.20	0.02
GFR < 60 ml/min/1.73 m^2^	1.56	1.11–2.89	0.04	0.95	0.93–0.97	<0.0001
Systolic BP > 140 mmHg	2.22	1.01–5.87	0.007	2.01	1.11–3.63	0.01
HbA1c (%)	1.77	1.30–2.41	0.0003	1.39	1.01–1.89	0.04
	*p* Value for model < 0.0001; χ^2^ = 38.47			*p* Value for model < 0.0001; χ^2^ = 42.41		

*BP* blood pressure, *GFR* glomerular filtration rate, *HbA1c* glycosylated haemoglobin

Mean HbA_1c_ in patients with HF was 8.6 ± 1.4 %.

At baseline, 78.5 % of the patients with HF were in the NYHA Class II and 21.5 % in the NYHA Class III. All patients with Class III were in subgroup C.

During the follow-up, none of the subjects died of HF, although transient exacerbation of symptoms (not requiring immediate hospitalization) was observed in 9.1 % of these patients.

## Discussion

Our study showed that patients with type 1 diabetes without any cardiovascular disorders have no evidence of MD and HF. MD and HF were observed only when diabetes coexisted with hypertension and significant CHD.

In the entire study group, the prevalence and incidence of both MD and HF were relatively low, although they were more common in patients with CHD compared with hypertensive subjects. Additionally, serum NT-proBNP levels were significantly higher in subjects with CHD compared with those who had only hypertension. This suggests a more advanced progression of HF due to myocardial ischaemia.

HFPEF was the most frequent manifestation of HF. No significant differences between sexes were observed.

To the best of our knowledge, the current study has been the first to report the prevalence and incidence of MD and HF in patients with type 1 diabetes. Similar studies, but focused on patients with type 2 diabetes, have already been published and attracted wide readership [1, 4, 6–10].

Numerous experimental studies describing the effect of acute hyperglycaemia (due to absolute insulin deficiency) on the development of myocardial damage suggested that elevated glucose levels may affect cardiac function [15–20]. However, the prevalence and incidence of MD and HF in our study cohort appear to be lower than expected. There are several possible explanations. First, all study subjects were on intensive insulin therapy, and concomitant treatment with statins, antihypertensive drugs, aspirin, and other medications was common. Second, glycaemic control, while not meeting the goals as defined by the Polish Diabetes Association (HbA_1c_ < 6.5 %) and the American Diabetes Association (HbA_1c_ < 7.0 %), was still satisfactory and similar to that reported in the EDIC study [21, 22]. Moreover, the study population was relatively young. Finally, in type 1 diabetes, other atherosclerotic risk factors including obesity, lipid abnormalities, or hypertension are less prevalent than in type 2 diabetes.

Although many investigators demonstrated some differences in the parameters of diastolic function and NT-proBNP levels between small groups of patients with type 1 diabetes without cardiovascular disorders and non-diabetic controls, none of them reported the prevalence and incidence rates of MD and HF [23–28]. Our study, similarly to some others, focused on normal diastolic function in type 1 diabetic patients [29, 30].

The most common abnormality in our study was diastolic manifestation of MD. It was observed at baseline in 15.6 % of hypertensive and coronary patients without HF. Zanchetti et al. reported that in the population diastolic dysfunction was observed in 26–46 % of elderly, hypertensive patients without HF (≥65 years) [31]. In our study, patients with hypertension and CHD were younger (mean baseline age, 57 years). Meanwhile, an early diagnosis of MD and treatment of predisposing disorders such as hypertension and CHD could potentially delay the onset of HF.

Several epidemiological studies have recently reported that HFPEF is observed in 50 % of the patients with HF, and the outcomes are similar to those seen in HFREF [14]. In our study, baseline HFPEF was observed in 85 % of the patients with HF and HFREF was observed in the remaining 15 %, due to the relatively low incidence of systolic dysfunction in coronary patients.

Interestingly, Lind et al. have recently published the results of their study in a large cohort of type 1 diabetic patients from the national Swedish diabetes registry. The authors reported an association between glycaemic control and the prevalence of HF [32]. They diagnosed HF mainly on the basis of clinical symptoms, while we also performed echocardiography and measured NT-proBNP levels. Additionally, we investigated MD and systolic and diastolic manifestations of HF.

Lind et al. considered all possible causes of HF including those observed in the general population (e.g. valvular diseases, atrial fibrillation). In contrast, we assessed the actual effect of diabetes alone or with hypertension and CHD on the prevalence and incidence of HF in type 1 diabetic patients. For this reason, the overall number of HF cases in the study by Lind et al. might be higher than in our study.

Similarly to Lind et al., we demonstrated that age, systolic blood pressure, CHD, duration of diabetes, and myocardial infarction were independent HF predictors. In the Swedish study, the incidence of HF was increased both in the lowest (<6.5 %) and highest (≥10.5 %) HbA_1c_ thresholds. In our study, HbA_1c_ level (mean value in HF patients, 8.6 %) was also an independent predictor of prevalent HF [32].

In a meta-analysis of randomised trials of intensive glycaemic control in patients with type 2 diabetes, intensive glycaemic therapy had no preventive effect on HF [33].

In contrast to the study by Lind et al., we did not observe that body mass index or smoking was independent predictors of HF in type 1 diabetes. Moreover, subjects with atrial fibrillation were excluded from our study (due to difficulties in assessing diastolic dysfunction). Furthermore, we determined that decreased glomerular filtration rate was an independent HF predictor. This particular finding was corroborated by other investigators who studied the general population [34].

The results of our study suggest that moderate hyperglycaemia alone does not exert a substantial adverse effect on myocardial function in type 1 diabetic patients without concomitant cardiovascular disorders. In type 1 diabetic patients with hypertension and CHD, the incidence of HF may be similar to that in the general population matched for age. Therefore, it seems quite likely that current pharmacotherapy targeting concomitant cardiovascular disorders exerts a cardioprotective effect by reducing the overall risk of cardiac complications.

Considering the high relative risk of major health burden associated with HF, it is important to investigate the potentially modifiable factors in the development of HF in type 1 diabetes.

The following study limitations should be considered. First, it was a large, prospective study that reflected a consecutive case series ascertained by the authors between 1999 and 2004. Because of the observational study design, it is impossible to draw any conclusion about the efficacy and safety of various types of modern therapy and their potential for preventing MD and HF in patients with type 1 diabetes. Moreover, not all age ranges were covered (the youngest patient at baseline was 20-year-old and the oldest was 65). Finally, there is no registry of HF patients in Poland so the actual number of patients with type 1 diabetes and concomitant HF may be underestimated.

In summary, in our patients with type 1 diabetes, MD and HF occurred only when diabetes coexisted with cardiovascular disorders affecting myocardial function. The prevalence and incidence of HF in diabetic subjects with hypertension and CHD were relatively low. While the cause of this observation remains uncertain, it could probably be explained, at least partially, by the cardioprotective effect of concomitant treatment.

## Abbreviations

A: Peak velocity of late left ventricular diastolic filling
A′: Late diastolic velocity at lateral mitral annulus
Ar: Pulmonary venous atrial reversal velocity
CHD: Coronary heart disease
DC: Diabetic cardiomyopathy
DT: E-wave deceleration time
E: Peak velocity of early left ventricular diastolic filling
E/A: Ratio of early and late left ventricular diastolic filling
E/E′: Ratio of E and E′
E′: Early diastolic velocity at lateral mitral annulus
HFPEF: Heart failure with preserved left ventricular ejection fraction
HFREF: Heart failure with reduced left ventricular ejection fraction
HbA1c: Glycosylated haemoglobin
LAVI: Left atrial volume index
LVDF: Left ventricular diastolic filling
LVEF: Left ventricular ejection fraction
NT-proBNP: N-terminal pro-B-type natriuretic peptide
S/D: Ratio of systolic and diastolic pulmonary forward flow
